# Hepatoprotective Effects of Swimming Exercise against D-Galactose-Induced Senescence Rat Model

**DOI:** 10.1155/2013/275431

**Published:** 2013-06-16

**Authors:** Chi-Chang Huang, Wen-Dee Chiang, Wen-Ching Huang, Chih-Yang Huang, Mei-Chich Hsu, Wan-Teng Lin

**Affiliations:** ^1^Graduate Institute of Sports Science, College of Exercise and Health Sciences, National Taiwan Sport University, Taoyuan 33301, Taiwan; ^2^Department of Food Science, Tunghai University, 181, Section 3, Taichung Kan Road, Taichung 40704, Taiwan; ^3^Graduate Institute of Athletics and Coaching Science, National Taiwan Sport University, Taoyuan 33301, Taiwan; ^4^Graduate Institute of Basic Medical Science, China Medical University, Taichung 40402, Taiwan; ^5^Department of Health and Nutrition Biotechnology, Asia University, Taichung 41354, Taiwan; ^6^Department of Sports Medicine, Kaohsiung Medical University, Kaohsiung 80708, Taiwan; ^7^Department of Hospitality, Tunghai University, Taichung 40704, Taiwan

## Abstract

This study investigates whether a 12-week swimming exercise training can prevent liver damage or senescence associated biomarkers in an experimental aging model in rats. Twenty-three male Sprague-Dawley rats were divided into four groups: vehicle treatment with sedentary control (C, *n* = 6), aging induction with sedentary (A, *n* = 6), vehicle treatment with swimming exercise (SW, *n* = 5), and aging induction with swimming exercise (A + SW, *n* = 6). Rats in groups A and AS received intraperitoneal d-galactose injections (150 mg/kg/day) for 12 weeks to induce aging. Rats in groups SW and A + SW were subjected to swimming exercise training for 12 weeks. Body weight, liver weight, epididymal fat mass, blood biochemistry, and liver pathology were performed at the end of the experiment. Hepatic senescence protein markers such as *β*-galactosidase, p53, and p21, as well as the inflammatory mediator, IL-6, were examined. The d-galactose-treated rats exhibited increases in AST and **γ**-GT plasma levels and *β*-galactosidase protein expression compared to the control group. Swimming exercise significantly reduced BW, epididymal fat mass, **γ**-GT activity, and p53, p21, and IL-6 protein levels compared to the aging group. These results suggest that a 12-week swimming exercise program suppresses senescence markers and downregulates inflammatory mediator in the liver tissues of d-galactose-induced aging rats.

## 1. Introduction

Chronic liver diseases are an important health issue worldwide, especially liver fibrosis [1]. Liver fibrosis results from prophase risks including chronic liver inflammation, fatty liver, and hepatic virus infection. The obese population has higher proportional fatty liver than the normal population. The fat accumulation in the liver causes abnormal protein expression of p53 and p21, which are important cell cycle regulator signaling pathways for senescence- or apoptosis-associated gene expressions [2]. Fatty liver increases pro-inflammatory cytokines expression and results in steatohepatitis, which can easily induce further liver fibrosis and cirrhosis processes. Currently, exercise is not only an integral component of a healthy lifestyle at weight loss, but also an independent benefit of exercise in non-alcoholic fatty liver disease and age-related loss in physical function in the elderly population has been demonstrated [3, 4]. Given the paucity of current treatment options on fatty liver and aging, exercise intervention could be expected to provide a low-cost therapy for disorders characterized by fatty liver and aging population.

d-Galactose is a kind of restoration hexoses that can provide energy and substances for the physiological requirements of macromolecular synthesis. d-galactose can react with the amine groups in free amino acids to cause nonenzymatic glycation, resulting in the accumulation of large advanced glycated protein end-products (AGE) [5]. These AGEs could combine with the receptors for advanced glycation end-products (RAGE) expressed on the peripheral vascular surface as well as internal organs such as the lungs, liver, and kidneys. This could induce tissue senescence, diabetes-associated inflammation, and immune system imbalance [6]. Excessive d-galactose administration produces a large number of free radicals, such as superoxide anions (^∙^O_2_^−^), reactive oxygen species (ROS), for example, hydrogen peroxide [7]. This oxidative stress causes mitochondrial dysfunction and destroys the cell structure and eventually the collapse of antioxidant mechanisms. Oxidative stress is the main factor that causes aging and even may shorten animal lifespan [8]. In d-galactose induced aging models the animals will produce oxidative stress, inflammation and malondialdehyde as well as a decrease in the total antioxidation status and superoxide dismutase [9, 10]. In previous studies, this d-galactose-induced aging model triggered the brain to reduce learning and memory [11] exhibited neuronal damage [12], as well as aging damage to the cardiovascular system, kidneys, and liver [9, 13, 14].

Oxidative pressure causes DNA lesions, p53 activation, and related genes expression is the main risk causing cellular senescence [15]. Cellular senescence is positively related to organ aging [16]. In liver tissues, cellular senescence could result ultimately in cirrhosis [17]. In the senescence process, several regulators arrest cell proliferation. p53 plays an important role in maintaining DNA integrity and keeps the cell cycle in the G1 phase [18]. p53 could be highly expressed under irradiation or free radical pressure to arrest the cell cycle from repairing DNA. If the repair system cannot completely fix mutations, the cells will be pushed into apoptosis [19]. p53 can activate the downstream p21 protein which inhibits the cyclin-CDK complex to stop cell proliferation [20].

The β-galactosidase (β-gal) gene has higher expression as well as the associated enzyme activity in aged cells [21]. This important indicator has a 3- to 5-fold higher expression in aged cells than in preaged cells. Studies have found that the liver tissue of patients with cirrhosis of the liver contains large amounts of β-gal [17]. β-Gal is often used as a biochemical marker to identify cells in the aging stage. Previous studies demonstrated that d-galactose could induce aging by increasing oxidative stress and reducing antioxidant defense mechanisms *in vivo*, causing liver aging and functional decline [22]. However, no studies have to date focused on the aging indicator, β-gal protein expression, or enzymatic activity in the liver under the d-galactose-induced aging model. Therefore, this study investigates the senescence marker protein levels associated with liver tissues and the preventive effects of regular exercise intervention in a d-galactose-induced aging rat model.

## 2. Materials and Methods

### 2.1. Chemicals and Antibodies

d-Galactose was purchased from Sigma-Aldrich Chemical Co. (St. Louis, MO, USA). Primary antibodies against β-gal from Abcam (MA, USA) were used in Western blotting. All other antibodies including p53, p21, IL-6, *α*-tubulin and second antibodies, and internal control *α*-tubulin were from Santa Cruz Biotechnology (Santa Cruz, CA, USA).

### 2.2. Animals, Treatment, and Swimming Protocol

Seven-week-old male Sprague-Dawley rats were purchased from BioLASCO (A Charles River Licensee Corporation, Yi-Lan, Taiwan). All animal experiments conformed to the guidelines of the Institutional Animal Care and Use Committee (IACUC) of Tunghai University. This study was conducted under the IACUC-98-27 protocol and approved by the IACUC ethics committee. The rats were raised for one-week before the experiments to adapt to the environment and diet. All animals were given a standard laboratory diet (no. 5001; PMI Nutrition International, Brentwood, MO, USA) and distilled water *ad libitum* and individually housed in a room maintained at 24 ± 2°C and humidity 55 ± 10% with a 12-h light-dark cycle during the one-week adaptation period. Afterward, the rats were randomly divided into four groups: (1) vehicle treatment with sedentary control (C, *n* = 6), (2) aging induction with sedentary control (A, *n* = 6), (3) vehicle treatment with swimming exercise (SW, *n* = 5), and (4) aging induction with a 12-week swimming exercise (A + SW, *n* = 6). The d-galactose was administrated by intraperitoneal (*i.p.*) injection with 150 mg/kg BW to groups A and A + SW. Equal volumes of vehicle (0.9% saline) were *i.p.* administered to groups C and SW. The d-galactose *i.p.* injections were administrated every day for 12 weeks to accelerate senescence induction. The swimming exercise protocol was conducted 5 times/week for 60 min/time for 12-week duration as previously described with some modifications [23]. The rats swam individually in a water sink with an area of 60 × 90 cm, 50 cm depth, with the water temperature kept at 35 ± 1°C.

### 2.3. Immunoblotting

Western blot analysis followed a previous report [24]. Protein content was measured using the Bradford method (Bio-Rad). Proteins were resolved using 5–20% gradient SDS-PAGE and then immunoblotted using an enhanced chemiluminescence assay (ECL; Perkin Elmer Life Science, Inc., USA) and image retrieval using the Fuji LAS-4000. The intensities were quantified using Alpha Ease FC software (Alpha Innotech software Inc., CA, USA).

### 2.4. TUNEL Assay

Terminal deoxynucleotidyl transferase-mediated dUTP nick end labeling (TUNEL) assay was used to detect *in situ* apoptotic cells according to the manufacturer's protocol (Chemicon/Millipore, Temecula, CA, USA). After deparaffinizing and rehydration pretreatments, the sections were immersed in 0.3% H_2_O_2_ for 30 minutes at room temperature to inactivate endogenous peroxidase activity. After rinsing with PBS, the sections were incubated with Proteinase K (2 mg/mL)/Tris buffer (10 mM, pH = 8) solution at room temperature for 15 min to enhance the permeability. After rinsing with PBS two times the sections were incubated in 0.1% Triton-100 (0.1% Sodium citrate) 4° for 8 minutes and then incubated with blocking buffer (Tris-HCl 0.1 M pH 7.5, 3% BSA and 20% normal bovine serum) for 1 h. The sections were rinsed in PBS twice, immersed in TUNEL Buffer (enzyme solution and label solution, 1 : 9) with 37°C in the dark and rinsed again with PBS. Samples were counterstained with DAPI to determine the nuclei localization.

### 2.5. Determination of Blood Biochemistry

Blood samples were collected from the rat abdominal aortas with indicated treatments after 12 weeks. The plasma was prepared by centrifugation at 1,500 ×g for 15 min at 4°C. The biochemical variables for liver injury indicators including AST, ALT, and *γ*-GT activities were analyzed by an autoanalyzer (Hitachi 7060, Hitachi, Tokyo, Japan).

### 2.6. Gross and Histological Liver Evaluation

Liver tissues were fixed in 10% formalin, then embedded in paraffin, and cut into 4 *μ*m thick slices. Tissue sections were stained with Hematoxylin and Eosin (H&E) or Masson trichrome and examined using a light microscope equipped with a CCD camera (BX-51, Olympus, Tokyo, Japan) as per our previous report [24].

### 2.7. Statistical Analysis

All data are represented as mean ± SEM. To evaluate the differences among the groups studied, data were analyzed using one-way ANOVA with the Statistical Analysis System (SAS Institute, Cary, NC, USA). *P* < 0.05 was considered statistically significant.

## 3. Results

### 3.1. Body Weight, Epididymal Fat Pad Mass, and Liver Weight

Morphological data from each experimental group are summarized in Table 1. There were no significant differences in the initial body and liws thedafdfver weights among groups C, A, SW and A + SW. Furthermore, d-galactose did not cause significant changes in the final body, liver and epididymal fat pad weights, compared to the control group. After 12-weeks swimming training there was a significant decrease in the final body weight in the SW and A + SW groups, by 11.5% (*P* = 0.0460) and 11.8% (*P* = 0.0331), respectively, compared to group A. There was also a significant decrease in the epididymal fat pad mass in the SW and A + SW groups by 47.0% (*P* = 0.0119) and 43.2% (*P* = 0.0180), respectively, compared to group A. We also observed that there was only a slight decrease in the liver weight in the A + SW group by 17.8% (*P* = 0.0558), compared to group A.

### 3.2. Swimming Exercise Protects Rats against d-Galactose-Induced Liver Dysfunction

**Figure 1:**
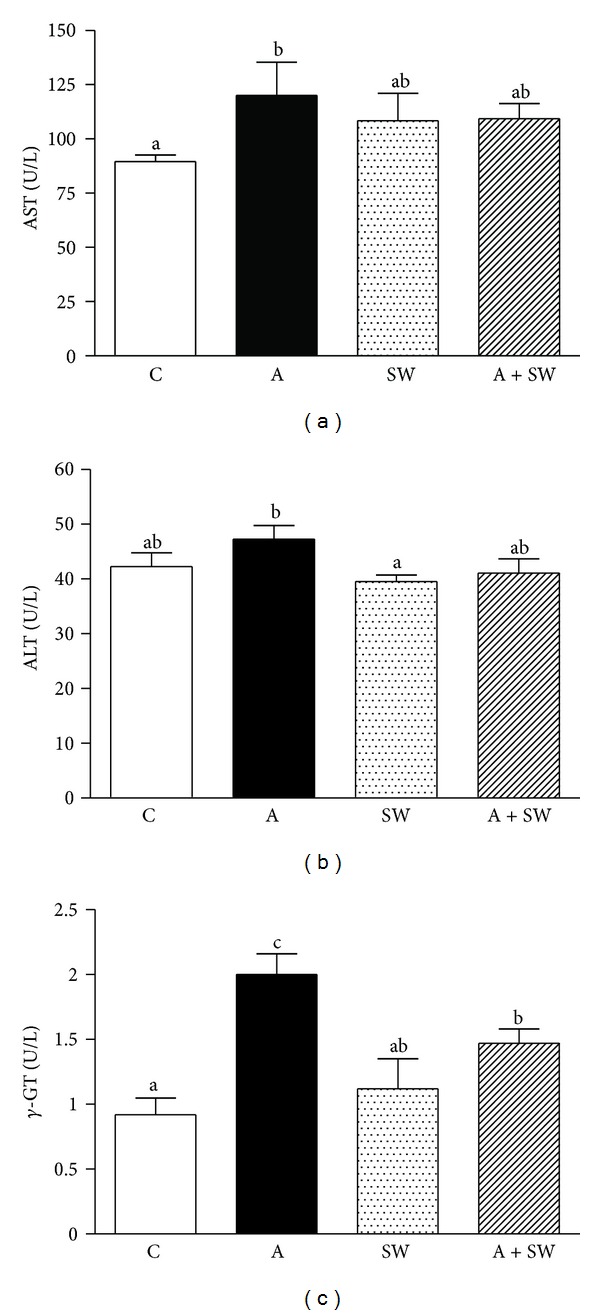
Swimming exercise training effect on plasma AST (a), ALT (b), and *γ*-GT (c) enzymatic activity in d-galactose-induced rat aging model. C: vehicle treatment with sedentary control (*n* = 6); A: aging induction with sedentary control (*n* = 6); SW: vehicle treatment with swimming exercise (*n* = 5); and A + SW: aging induction with a 12-week swimming exercise (*n* = 6). Data represent mean ± SEM. Bars marked with different letters are significantly different from each other (*P* < 0.05).

Figure 1 shows that the plasma AST activity in the groups C, A, SW, and A + SW were 88.4 ± 2.7, 120.0 ± 15.4, 108.4 ± 12.7, and 109.3 ± 7.0, respectively. There was only a significant increase in the AST level in group A by 1.4-fold (*P* = 0.0421), compared to the control group. The plasma ALT activity in groups C, A, SW, and A + SW were 42.3 ± 2.5, 47.3 ± 2.5, 39.5 ± 1.2, and 41.1 ± 2.6, respectively. There was only a significant decrease in the ALT level in the SW group by 16.4% (*P* = 0.0351) and a slight decrease in the A + SW group by 13.1% (*P* = 0.0729), respectively, compared to group A. 

The plasma *γ*-GT activity in groups C, A, SW, and A + SW were 0.92 ± 0.13, 2.00 ± 0.16, 1.12 ± 0.23, and 1.47 ± 0.11, respectively. Group A showed a significant increase in the *γ*-GT level by 2.2-fold (*P* < 0.0001), compared to the control group. After 12-week swimming training, we found that the plasma *γ*-GT levels significantly decreased in groups SW and A + SW by 44.0% (*P* = 0.0010) and 26.7% (*P* = 0.0234), respectively, compared to group A. 

**Figure 2:**
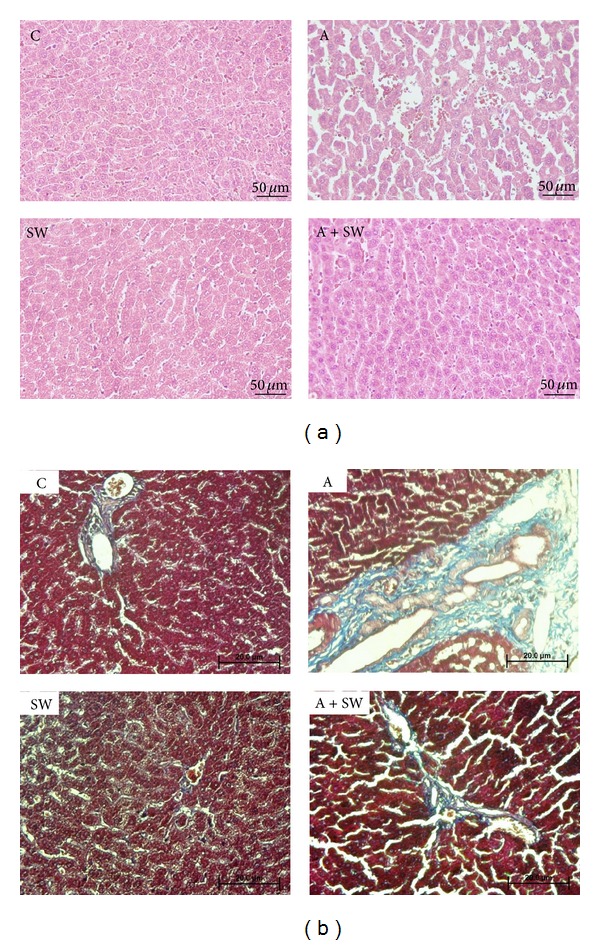
(a) Swimming exercise training effect on liver pathology in d-galactose induced rat aging model. C: vehicle treatment with sedentary control (*n* = 6); A: aging induction with sedentary control (*n* = 6); SW: vehicle treatment with swimming exercise (*n* = 5); A + SW: aging induction with a 12-week swimming exercise (*n* = 6). Representative hepatic sections of rats from each group stained with H&E. Scale bars, 50 *μ*m. (Magnification, 200x). (b) Swimming exercise training effect on liver fibrosis in d-galactose induced rat aging model. C: vehicle treatment with sedentary control (*n* = 6); A: aging induction with sedentary control (*n* = 6); SW: vehicle treatment with swimming exercise (*n* = 5); A + SW: aging induction with a 12-week swimming exercise (*n* = 6). Histological sections of the liver from each group stained with Masson trichrome (fibrosis: blue color). Scale bars, 200 *μ*m. (Magnification, 200x).

The histology of hepatic structure is shown in Figure 2(a). To evaluate whether d-galactose treatment could induce aging-like changes in the liver, the pathology examination of liver tissues from rats in each group was measured. The hepatic cords arranged loosely; dilatation of sinusoid, hepatocytes vacuolation, and multi focal necrosis were observed in the d-galactose-treated animals compared to those in the control group. According to the grading and score of lesions, these pathological changes were almost completely ameliorated by 12-weeks swimming training (Table 2).

### 3.3. Swimming Exercise Protects Rats against d-Galactose-Induced Liver Fibrosis

d-Galactose also caused significant interstitial collagen deposition, as demonstrated by Masson's trichrome staining. This fibrosis was also significantly reduced in d-galactose-treated rats subjected to swimming training for 12 weeks (Figure 2(b) and Table 2).

### 3.4. Swimming Exercise Protects Rats against d-Galactose-Induced Liver Apoptosis

**Figure 3:**
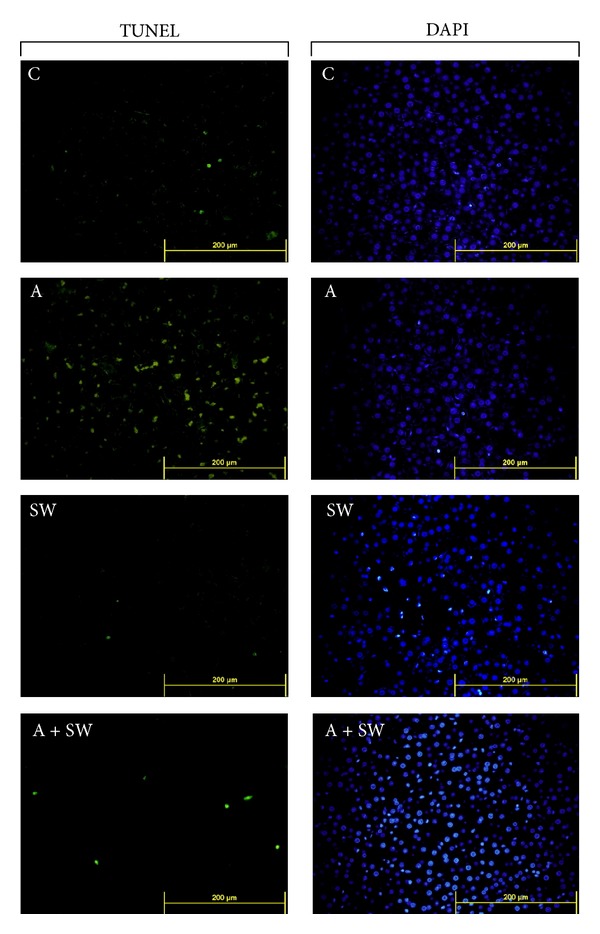
Swimming exercise training effect on liver apoptosis in d-galactose-induced rat aging model. C: vehicle treatment with sedentary control (*n* = 6); A: aging induction with sedentary control (*n* = 6); SW: vehicle treatment with swimming exercise (*n* = 5); and A + SW: aging induction with a 12-week swimming exercise (*n* = 6). Localization of apoptotic cells using TUNEL assay in liver tissues from each group. The figures show hepatic cells stained with fluorescein-dUTP (green) and counterstained with DAPI (blue). Scale bars, 200 *μ*m. (Magnification, 200x).

As shown in Figure 3, d-galactose caused significant apoptosis in the rat liver tissues, as demonstrated by TUNEL assay, compared with the control group (C). This apoptosis was also significantly reduced in d-galactose-treated rats subjected to swimming training for 12 weeks.

### 3.5. Effects of Swimming Exercise on Protein Expression of Hepatic β-Gal, p53, p21, and IL-6 in d-Galactose-Treated Rats

**Figure 4:**
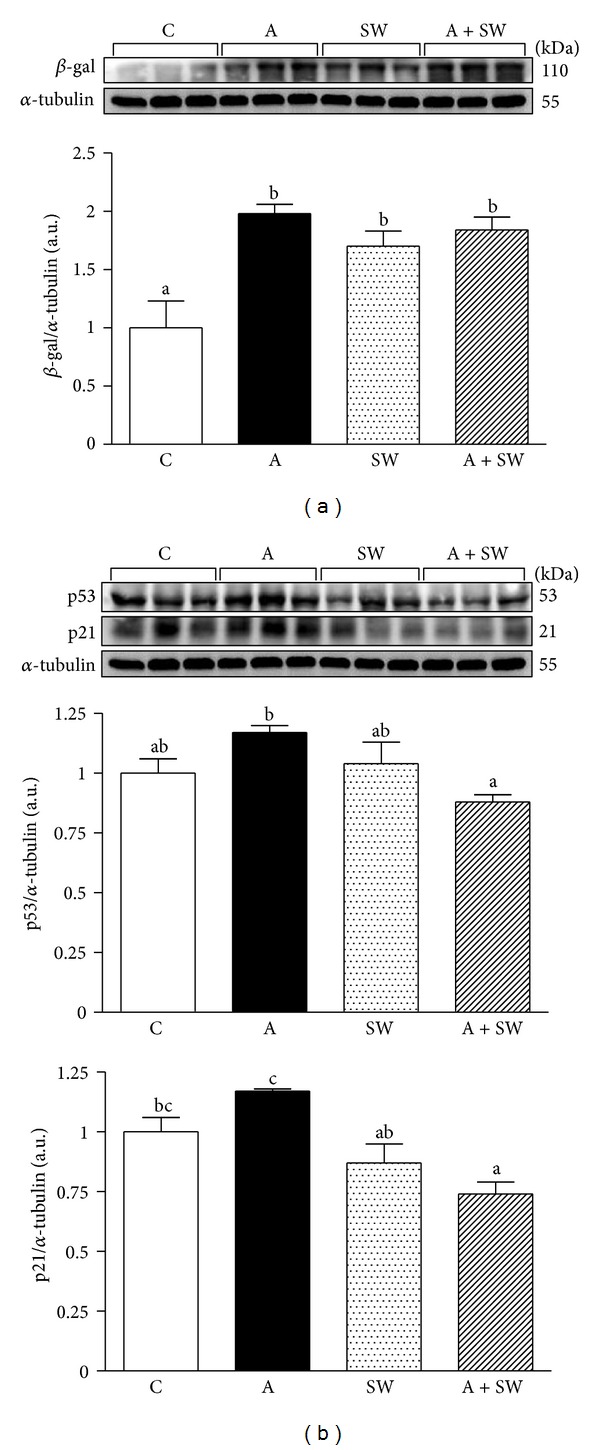
(a) Swimming exercise training effect on β-galactosidase expression of liver tissues in d-galactose-induced rat aging model. C: vehicle treatment with sedentary control (*n* = 6); A: aging induction with sedentary control (*n* = 6); SW: vehicle treatment with swimming exercise (*n* = 5); and A + SW: aging induction with a 12-week swimming exercise (*n* = 6). Data represent mean ± SEM. Bars marked with different letters are significantly different from each other (*P* < 0.05). (b) Swimming exercise training effect on p53 and p21 proteins expression of liver tissues in d-galactose induced rat aging model. C: vehicle treatment with sedentary control (*n* = 6); A: aging induction with sedentary control (*n* = 6); SW: vehicle treatment with swimming exercise (*n* = 5); and A + SW: aging induction with a 12-week swimming exercise (*n* = 6). Data represent mean ± SEM. Bars marked with different letters are significantly different from each other (*P* < 0.05).

Figure 4(a) shows that the hepatic β-gal protein expression in the groups C, A, SW, and A + SW were 1.00 ± 0.23, 1.98 ± 0.08, 1.70 ± 0.13, and 1.84 ± 0.11, respectively. We found that the β-gal levels of liver tissues significantly increased in groups A (*P* = 0.0014), SW (*P* = 0.0093) and A + SW (*P* = 0.0035) by 2.0-, 1.7- and 1.8-fold, respectively, as compared to the group C. 

Figure 4(b) shows that the hepatic p53 protein expression in groups C, A, SW, and A + SW were 1.00 ± 0.06, 1.17 ± 0.03, 1.04 ± 0.09, and 0.88 ± 0.03, respectively. There was a slight increase in the p53 level in the A group compared to the control group (*P* = 0.0842). After 12-week swimming training we found a significant decrease in the p53 expression in liver tissues in groups A + SW by 24.9% (*P* = 0.0090) compared to group A. The hepatic p21 protein expression in groups C, A, SW, and A + SW were 1.00 ± 0.06, 1.17 ± 0.01, 0.87 ± 0.08, and 0.74 ± 0.05, respectively. After 12-week swimming training there was a significant decrease in the p21 expression in liver tissues in groups SW, and A + SW by 25.3% (*P* = 0.0065) and 36.8% (*P* = 0.0007), respectively, compared to group A.

**Figure 5:**
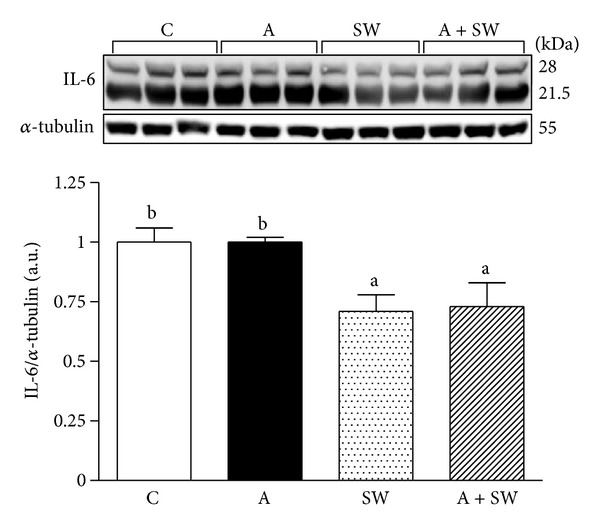
Swimming exercise training effect on IL-6 expression in liver tissues in d-galactose induced rat aging model. C: vehicle treatment with sedentary control (*n* = 6); A: aging induction with sedentary control (*n* = 6); SW: vehicle treatment with swimming exercise (*n* = 5); A + SW: aging induction with a 12-week swimming exercise (*n* = 6). Data represent mean ± SEM. Bars marked with different letters are significantly different from each other (*P* < 0.05).

As shown in Figure 5, the hepatic IL-6 protein expression in the groups C, A, SW, and A + SW were 1.00 ± 0.06, 1.00 ± 0.02, 0.71 ± 0.07, and 0.73 ± 0.10, respectively. After 12-week swimming training we found a significant decrease in the IL-6 expression in liver tissues in groups SW and A + SW by 28.6% (*P* = 0.0182) and 27.4% (*P* = 0.0225), respectively, compared to groups C and A. 

## 4. Discussion

β-Gal has higher enzyme activity and expression in aged organs, and it is also an important indicator in aged cells [21]. The d-galactose-induced aging model could increase the oxidative pressure and inflammation, causing senescence injury. This senescence-induced model could result in a decline in cognitive function in the brain and cardiovascular damage [11, 13], but there are no studies focused on the liver β-gal activity on long-term d-galactose administration. This study showed a 12-week d-galactose induction of A and A + SW groups could significantly induced higher β-gal expression, which means that liver is in senescence status. 

An increase in body fat often occurs in old age. The obese have a higher chance of suffering from nonalcoholic steatohepatitis than those with standard weight [25]. Therefore, obesity with age increases the risk for liver fibrosis. However, comparison between the C and A groups showed no significant difference in body weight and epididymal fat at the end of the experiment. The occurrence of natural aging could be regulated by many factors or physiological effects [26], including glycation oxidative injury from this d-galactose induction model. This may explain why the body weight and fat did not increase, which is different from natural aging.

The body weight and epididymal fat increases with aging-related obesity are due to accumulated liver fat content, resulting in liver weight increase [27]. It is clear that metabolic dysfunction of both carbohydrate and fat can be attributed to lipid accumulation and mitochondrial dysfunction and leads to obesity and type 2 diabetes. In contrast, exercise enhances mitochondrial performance, favoring tighter coupling between β-oxidation and the TCA cycle. A growing body of evidence has demonstrated that exercise can upregulate the transcriptional coactivator PGC-1 alpha (PGC-1*α*) and leads to enhanced lipid metabolism, improved mitochondrial function, and mitochondrial biogenesis [28]. We found in this study that 12-week swimming exercise could significantly decrease the body weight and liver weight in the A + SW group compared to the A and SW groups, which both exhibited significantly lower fat content than the C group. This data demonstrates that the 12-week exercise program improved aging-related obesity and effectively controlled the body weight.

The cell cycle is regulated by important regulators such as p53 and p21 proteins which activate deterioration in the senescence process [29]. p53 activation can activate downstream p21 to maintain cell cycle arrest for DNA repair [30]. If the damage cannot be completely repaired, the cell will progress into apoptosis programs to maintain homeostasis [19]. Obese animals have higher body fat and exhibit higher β-gal enzyme activity and p53 expression [15]. In this senescence induced study, the fat weight, β-gal activity, p53 and p21 expression were significantly higher than those C group. This represents that hepatocytes developed into the senescence pathway in this induction model. After exercise intervention, the p53 and p21 expression in the A + SW group was inhibited, preventing normal proliferation and replication processes.

Hepatocytes in fatty liver or fat infiltration will induce inflammation by cytokine secretion of IL-6 and further process into liver fibrosis lesions [31]. Therefore, TNF-*α* and IL-6 secretion inhibition was considered to be able to reduce liver fibrosis [32, 33]. The previous studies showed that exercise training could have anti-inflammatory effects by decreasing the C-reactive protein (CRP), TNF-*α*, and IL-6 productions [34, 35]. We found that the proinflammation cytokine, IL-6 and protein levels of liver tissues were inhibited by the swimming exercise. Therefore, the swimming exercise may have a beneficial effect in preventing liver fibrosis via inflammatory response reduction in d-galactose-induced aging animals.

This study found that the proinflammation cytokines in group A were not significantly higher than those in the control group. We suppose that the d-galactose-induced inflammation maybe a kind of blood circulating inflammation with less effect on local organ inflammation. Aging associated proteins could directly regulate liver hepatocyte apoptosis or senescence in this induction model, not by inflammation effects secreted by aging cells [16].

Histologic results showed that the hepatocytes were arranged with complete structural stability in the C and SW groups. Cellular vacuolation and ballooning degeneration also did not appear. In the A group, the hepatic cords were in a messy loose arrangement; dilatation of sinusoid, hepatocytes vacuolation and multifocal necrosis were observed [36]. The liver tissue hepatic structure in group A + SW was arranged more regularly than that of group A. Twelve weeks of d-galactose-induced aging in this study resulted in loose hepatocyte arrangement, mild hepatocytes vacuolation, and punctate necrosis. After 12 weeks of swimming exercise training, the hepatocyte structural integrity showed no irregular collagen hyperplasia inflammation reaction in the cell. 

The liver collagen fiber distribution was evaluated using Masson's trichrome staining, which gave the sample a blue purple color. The staining results showed the peripheral liver vascular tissue with little collagen fibers in the C and SW groups. The tissue interval contained less collagen fiber with less blue-violet distribution. The A group showed more collagen fiber staining in perivascular area and also in the liver tissue interval, surrounding the liver tissue forming irregular pseudo-lobules presenting a hepatic fibrosis state. In the TUNEL assay, the A group also showed an average number of apoptotic cells that was significantly higher than that in the C group. The swimming exercise intervention results for (A + SW group) compared to group A showed less collagen fiber accumulation and improved pseudolobules formation within the liver tissue. A twelve-week swimming intervention was found to reduce the d-galactose-induced collagen fiber formation in liver tissues. 

In the biochemical results, we found that AST was significantly increased in the swimming intervention group compared to the C group but the ALT did not show any difference between the groups. However, AST is not specifically produced by the liver and is also used as an injury indicator for cardiac or skeletal muscle [37, 38]. Previous studies showed that exercise training could induce LDH and CK activity and also AST and ALT from muscles [39, 40]. The aged group had higher AST and ALT activities compared to the young group in the same liver injury model [41]. The increase in AST may be caused by exercise intervention, not by liver disease including inflammation, fatty liver, or fibrosis. The *γ*-GT activity in this senescence induction model was significantly increased after 12 weeks of d-galactose induction, and the increase in *γ*-GT activity is a risk factor for chronic liver disease formation.

In the results from this d-galactose-induced, senescence rat model showed that the liver could be induced to express the senescence indicator, β-gal, and pathological appearance of liver specimens from d-galactose-treated animals. The swimming exercise intervention could prevent the liver pathological changes associated with age-related liver injury, as well as the inflammatory cytokine and cell cycle-associated regulators involved in senescence progression. In a future study, we could investigate the preventive or therapeutic effects of different types of exercise using this chemical-induced aged rat model. Many factors, both extrinsic and intrinsic, cause different tissues to age; therefore, the natural senescence process should be a more reliable model to study the preventive or therapeutic effects of exercise intervention. In parallel, more research is also needed to better understand the different markers affected by the specific aging tissue progressions and the role of exercise in the management of target tissue senescence.

## Figures and Tables

**Table 1:** General characteristics of the experimental groups.

Characteristics	C	A	SW	A + SW
Initial BW (g)	234 ± 3	232 ± 4	234 ± 4	235 ± 2
Final BW (g)	527 ± 17^ab^	549 ± 20^b^	486 ± 30^a^	484 ± 15^a^
Epididymal fat pad (g)	4.60 ± 0.49^b^	5.25 ± 0.47^b^	2.78 ± 0.43^a^	2.98 ± 0.39^a^
Liver (g)	13.2 ± 0.6	13.9 ± 0.9	12.6 ± 1.2	11.7 ± 0.4

Values are means ± SEM. C: vehicle treatment with sedentary control (*n* = 6); and A: aging induction with sedentary control (*n* = 6); SW: vehicle treatment with swimming exercise (*n* = 5); A + SW: aging induction with a 12-week swimming exercise (*n* = 6). Data represent mean ± SEM. Values that have a different superscript letter (a, b) differ significantly with each other (*P* < 0.05).

**Table 2:** Effects of swimming exercise training on the histological features of liver tissues.

Parameters	C	A	SW	A + SW
H&E stain				
Arrangement of hepatic cords	0 ± 0	1.0 ± 0.0	0 ± 0	0 ± 0
Dilatation of sinusoid	0 ± 0	1.2 ± 0.1	0 ± 0	0.4 ± 0.1*
Hepatocyte vacuolation	0 ± 0	1.6 ± 0.2	0 ± 0	0.5 ± 0.1*
Multifocal necrosis	0 ± 0	0.5 ± 0.1	0 ± 0	0 ± 0
Masson's trichrome				
Cholangio fibrosis	0 ± 0	1.3 ± 0.2	0 ± 0	0.3 ± 0.0*

The grading and score with definition of lesions: 0 = no lesions; 1 = mild, 2 = moderate; and 3 = severe. Arrangement of hepatic cords and dilatation of sinusoid: 0 = normal; 1 = perivenular; 2 = extensive to midzone; and 3 = diffuse. Hepatocyte vacuolation: 0 = no vacuolation; 1 = 3–9 hepatocyte vacuolations; 2 = 10–30 hepatocyte vacuolations; and 3 = more than 30 hepatocyte vacuolations. Multi focal necrosis: 0 = no necrosis; 1 = 1–3 foci; 2 = 4–10 foci; 3 = more than 11 foci. Cholangio fibrosis: 0 = normal portal area; 1 = 3-4 portal areas; 2 = 5–8 portal areas; and 3 = more than 9 portal areas. Values are expressed as means ± SEM. C: vehicle treatment with sedentary control (*n* = 6); A: aging induction with sedentary control (*n* = 6); SW: vehicle treatment with swimming exercise (*n* = 5); and A + SW, aging induction with a 12-week swimming exercise (*n* = 6). *P < 0.05 compared to the group A.
